# Five Year Incidence of Visual Field Loss in Adult Chinese. The Beijing Eye Study.

**DOI:** 10.1371/journal.pone.0037232

**Published:** 2012-05-18

**Authors:** Ya Xing Wang, Liang Xu, Xiu Ying Sun, Yang Zou, Hai Tao Zhang, Jost B. Jonas

**Affiliations:** 1 Beijing Institute of Ophthalmology, Beijing Tongren Hospital, Capital Medical University, Beijing, China; 2 Department of Ophthalmology, Medical Faculty Mannheim of the Ruprecht-Karls-University Heidelberg, Germany; Massachusetts Eye & Ear Infirmary, Harvard Medical School, United States of America

## Abstract

**Purpose:**

To describe the cumulative 5 year incidence of visual field loss in adult Chinese in Greater Beijing.

**Methods:**

The Beijing Eye Study 2006 included 3251 subjects (mean age 60.4±10.1 years) who had participated in the Beijing Eye Study 2001 and returned for re-examination. All participants underwent a comprehensive eye examination, including visual field test by frequency doubling threshold perimetry. An abnormal visual field was defined as reduced sensitivity in at least one test location. Incident visual field loss was defined as a change in visual field from normal at baseline to abnormal at follow-up.

**Results:**

An incident visual field loss was detected in 273 eyes (4.3±0.5%) /235 subjects (7.3±0.5%). It was significantly associated with higher age (*P* = 0.001), higher intraocular pressure (*P*<0.001), and higher fasting blood glucose concentration (*P* = 0.019). Considering only eyes (n = 140) with a detected cause for visual field loss, the most frequent causes were cataract (68 (48.6%) eyes) followed by glaucoma (23 (16.4%) eyes), diabetic retinopathy (13 (9.3%) eyes), age-related macular degeneration (10 (7.1%) eyes), and myopic degenerative retinopathy (9 (6.4%) eyes). For 133 (48.7%) eyes with a visual field loss, the cause for the VFL remained unclear.

**Conclusions:**

The 5-year incidence of visual field loss was 4.3±0.5% per eye or 7.3±0.5% per subject. It increased significantly with age, intraocular pressure, and fasting blood glucose level. Major causes for the incidence of visual field loss were cataract, glaucoma and diabetic retinopathy.

## Introduction

Knowledge about prevalence and reasons of impaired visual acuity and visual field defects is of importance for public health policies and helps to better understand the clinical background of eye diseases. While numerous previous studies have examined the prevalence of impaired visual acuity in many ethnic groups around the globe [1]–[6], and while fewer studies have evaluated the incidence of visual acuity loss [7]–[12], relatively few studies were mainly focused on the prevalence of visual field defects [13]–[21], and only rarely, an investigation addressed the incidence of visual field loss (VFL) [22]–[25]. Since the paracentral and peripheral visual field is of utmost importance for the quality of vision, knowledge about the incidence of VFL may, however, be similarly important as the knowledge about the incidence of visual acuity loss. The scarcity of information on the incidence of VFL holds true in particular for China with the world's largest population. Knowledge about incident visual field loss may be of particular importance for the Chinese population since it undergoes pronounced demographic changes with an ageing of the population, so that medical monitoring and care for senior citizens will become increasingly important. It was, therefore, the purpose of our study to assess the incidence of VFL as determined by frequency doubling threshold perimetry in the follow-up of the Beijing Eye Study from 2001 to 2006, and to inquire the causes for incident VFL.

## Methods

### Ethics Statement

The Medical Ethics Committee of the Beijing Tongren Hospital approved the study protocol, and all participants gave informed consent, according to the Declaration of Helsinki.

The population-based Beijing Eye Study was first performed in the year 2001, and the follow up study was carried out 5 years later in 2006. At baseline, 4439 subjects out of 5324 eligible subjects were included, corresponding to a response rate of 83.4%. The study was divided into a rural part (1973 subjects; 1143 women) and an urban part (2466 subjects; 1362 women). The mean age was 56.2±10.6 years (median: 56 years; range: 40–101 years). At baseline and at the follow-up examination, a comprehensive eye examination was carried out including visual acuity assessment, perimetry, noncontact tonometry (CT-60 computed tonometer, Topcon Ltd., Tokyo, Japan), slit-lamp examination of the external eye and anterior segment, and photography of the lens (Neitz CT-R camera, Neitz Instruments Co., Tokyo, Japan) and ocular fundus (CR6-45NM, Canon Inc. Tokyo, Japan). The visual field examinations were performed by frequency-doubling perimetry using the screening program C-20-1 (Zeiss-Humphrey, Dublin, California, USA) [24]. An examination result was considered valid if the rate of false positive results and the rate of fixation loss were equal to or lower than 0.33. We defined an abnormal frequency doubling perimetry as at least one location of reduced sensitivity of any level. An incident VFL was defined to be present if the visual field was normal in the examination of the year 2001 and abnormal in the test performed in 2006. Past history of ocular and systemic diseases, the socioeconomic background and general aspects such as level of education, family income, smoking and alcohol consumption were assessed in a questionnaire. At the follow up examination in 2006, we additionally measured body weight and height and arterial blood pressure, and we took fasting blood samples. The concentrations of glucose, cholesterol, low-density lipoproteins and high-density lipoproteins were measured. The study was previously described in detail [27]–[29].

For the determination of the principle cause of incident VFL, the photographs of the lens and fundus taken in 2001 and 2006 were assessed by an experienced ophthalmologist (YXW). If multiple diseases were detected in an eye, the primary cause for visual impairment or blindness was determined by a panel (YXW, LX, JBJ). In a similar manner, if the initial reviewer could not decide on the cause for the VFL or if the cause of VFL was unknown, the panel of 3 ophthalmologists worked together to decide on the etiology.

Cataract was considered to be the major reason for visual field loss, if the fundus appeared to be without major abnormality and if the lens photographs in 2006 in contrast to 2001 showed a nuclear lens opacification of grade “3” or higher or if considerable cortical or posterior subcapsular opacifications were present [27]. Degenerative myopic retinopathy was considered to be the major cause for an incident VFD, if the macular region showed myopic atrophic changes, if myopic refractive error exceeded –6 diopters, and if the optic disc appeared normal [30]. The optic disc was considered to be normal if the neuroretinal rim followed the so-called ISNT-rule, and if the visibility of the retinal nerve fiber layer did not show localized defects or a marked segmental loss, as far as the bright fundus in highly myopic eyes allowed an examination of the retinal nerve fiber layer [31]. Non-glaucomatous optic nerve damage was assumed to be the reason for visual field loss, if the neuroretinal rim fulfilled the ISNT-rule, if the neuroretinal rim was pale, if the visibility of the retinal nerve fiber layer was reduced, and if the retinal arteriole diameter was decreased [32]. For the definition of age-related macular degeneration, the classification and grading system recommended by the international Age-Related Maculopathy epidemiological study group was used [33]. For the definition of diabetic retinopathy, the Early Treatment of Diabetic Retinopathy Study (ETDRS) criteria were applied [34]. The minimum criterion for diagnosis of diabetic retinopathy was the presence of at least one definite microaneurysm. Retinal branch vein occlusion was defined as edematous and hemorrhagic changes or partially occluded veins with or without collaterals in one of the four fundus regions [35]. If no obvious morphological ocular abnormality was found responsible for the incident VFL, we termed it an incident VFL of unknown cause.

Statistical analysis was performed using a commercially available statistical software package (SPSS for Windows, version 20.0, SPSS Inc., Chicago, IL). The frequency of VFL was given as mean±standard error and as 95% confidence intervals (CI). The mean values of all other parameters were given as mean±standard deviation. Multiple logistic regression analysis was used to analyze the factors related to the incidence of VFL.

## Results

At the baseline examination in the year 2001, frequency doubling perimetry test results were available for 8719 eyes of 4369 subjects. Out of these eyes, the rate of false positive results was higher than 0.33 in 57 (0.7%) eyes which were excluded from further statistical analysis. Out of the whole study population, the rate of fixation loss was higher than 0.33 in 75 (0.9%) eyes. Out of these 75 eyes, 28 (37.3%) eyes had a rate of false positive answers higher than 0.33. For further analysis, all eyes (n = 104) with a rate of false positive answers higher than 0.33 and all eyes with a rate of fixation loss higher than 0.33 were excluded so that the final study population at baseline examination eventually consisted of 4349 patients (8615 eyes). A bilateral examination was available for 4267 subjects, and 83 subjects had unilateral frequency doubling perimetry. There were 1913 (44.0%) subjects from the rural region, and 2436 (56.0%) subjects from the urban region. There were 13 (0.2%) aphakic eyes, and 59 (0.7%) pseudophakic eyes. The details were published previously [16], [19].

Among the 4439 subjects who participated in the baseline study in 2001, 3251 (73.2%) returned for the follow-up study in 2006. The remaining subjects had either died during the follow-up (143 subjects; 3.2%), or did not re-participate (1045 subjects; 23.5%). From the 4349 subjects (8615 eyes), for whom a reliable visual field examination was available in 2001, 3219 (74.0%) subjects participated in the follow-up examination. In the latter, reliable visual field examinations were available for 6401 eyes of 3211 participants. For 22 eyes out of these 6401 eyes, reliable perimetric examinations for 2001 were not available, so that the study eventually included 6379 eyes (3200 participants) for which reliable visual field examinations were available for both 2001 and for 2006.

An incident VFL was found in 273 eyes (4.3±0.5%; 95%CI: 3.8, 4.8) or 235 participants (7.3±0.5% (93%CI: 6.4, 8.2)). There were 38 subjects (1.2±0.2%; 95%CI: 0.8, 1.6) who showed bilateral incident VFL. The group of subjects with an incident VFL as compared with the group of subjects with normal visual fields in both 2001 and 2006 revealed that subjects with an incident VDL were significantly older (*P*<0.001), had higher systolic blood pressure (*P* = 0.003) and higher fasting blood glucose concentrations (*P*<0.001), and had a lower visual acuity (*P*<0.001), a more myopic refractive error (*P* = 0.003), and higher intraocular pressure (*P*<0.001) (univariate analysis) (Table 1). The association between the incidence of VFL and age was confirmed in a Chi-square test for trend (*P*<0.001). The five-year incidence of VFL was 1.2±0.2% (95%CI: 0.7, 1.6) per eyes or 2.0±0.4% (95%CI: 1.2, 2.8) per subjects in the age group of 40 to 49 years, 3.5±0.4% (95%CI: 2.6, 4.3) per eye or 6.1±0.8% (95%CI: 4.6, 7.7) per subject for the age group from 50 to 59 years, 6.9±0.6% (95%CI: 5.7, 8.0) per eye or 11.3±1.0 (95%CI: 9.3, 13.4) per subjects for the age group from 60 to 69 years, and 10.6±1.3% (95%CI: 8.0, 13.2) per eye or 19.2±2.3% (95%CI: 14.5, 23.8) per subject for the age group of 70+years, respectively.

**Table 1:** Association between incident visual field loss and general or ocular parameters in the Beijing Eye Study using univariate analysis.

	Regression Coefficient	P-Value	Odds Ratio	95% CI
Age (Years)	0.08	<0.001	1.08	1.07, 1.10
Gender (Men/Women)	0.14	0.267	1.15	0.90, 1.47
Urban/Rural Region of Habitation	0.09	0.489	1.09	0.85, 1.39
Level of Education (0–5)	−0.14	0.064	0.87	0.75, 1.01
Refractive Error (Diopters)	−0.07	0.003	0.93	0.89, 0.98
Family Income	0.00	0.217	1.00	1.00, 1.00
Intraocular Pressure (mmHg)	0.07	<0.001	1.07	1.04, 1.10
Cup/Disc Diameter Ratio	0.43	0.386	1.53	0.59, 4.01
Vertical Optic Disc Diameter (mm)	−0.06	0.853	0.94	0.50, 1.77
Body Height (cm)	−0.01	0.078	0.98	0.96, 1.00
Body Weight (kg)	−0.02	0.849	1.00	0.98, 1.01
Body Mass Index (kg/m^2^)	0.02	0.314	1.02	0.98, 1.07
Systolic Blood Pressure (mmHg)	0.01	0.003	1.01	1.00, 1.02
Diastolic Blood Pressure (mmHg)	−0.01	0.239	0.99	0.98, 1.01
Fasting Blood Glucose (mmol/L)	0.13	<0.001	1.14	1.07, 1.22

A binary logistic regression analysis, with the incidence of VFL as dependent parameter and age, systolic blood pressure, fasting blood glucose levels, visual acuity, refractive error and intraocular pressure as independent variables, showed that the incidence of a VFL remained to be significantly associated with higher age (*P* = 0.001), higher intraocular pressure (*P*<0.001), and higher fasting blood glucose concentrations (*P* = 0.019) (Table 2).

**Table 2:** Associations between incident visual field loss and general or ocular parameters in the Beijing Eye Study 2001/2006, using a binary logistic regression analysis.

	Regression Coefficient	P-Value	Odds Ratio	95% CI
Age (Years)	0.09	0.001	1.10	1.07, 1.12
Refractive Error (Diopters)	−0.06	0.057	0.94	0.88, 1.00
Intraocular Pressure (mmHg)	0.09	<0.001	1.10	1.05, 1.14
Systolic Blood Pressure (mmHg)	0.00	0.74	1.00	0.99, 1.01
Fasting Blood Glucose (mmol/L)	0.09	0.019	1.09	1.01, 1.18

The most frequent cause for incident VFL was cataract (68 (24.9%) eyes (or 48.6% of the eyes with a detected cause for VFL)), followed by glaucoma (23 (8.4%) eyes (or 16.4% of the eyes with a detected cause for VFL)), diabetic retinopathy (13 (4.8%) eyes (or 9.3% of the eyes with a detected cause for VFL)), age-related macular degeneration (10 (3.7%) eyes (or 7.1% of the eyes with a detected cause for VFL)), and myopic degenerative retinopathy (9 (3.3%) eyes (or 6.4% of the eyes with a detected cause for VFL)) (Table 3). Stratifying the study population by age revealed that glaucoma was the leading cause for incident VFL in the age group from 45 to 54 years (4 out 11 eyes (36.4%; 95%CI: 7.9, 64.8)), and that cataract became the most frequent cause for incident VFL in the age group of 55 or more years (Table 4). For 133 (48.7%) eyes, a reason for the incident VFL was not detected. The subjects without a detected cause for an incident VFL as compared with the subjects with a detected cause were significantly younger (66.5±8.9 years versus 69.3±9.3 years; *P* = 0.01) and had a higher best corrected visual acuity (*P*<0.001). If the subjects without a detected cause for an incident VFL were compared with the subjects with a normal visual field, they were significantly older (66.5±8.9 years versus 59.5±9.7 years; *P*<0.001) and had a lower visual acuity (*P*<0.001).

**Table 3:** Causes for incident visual field loss in the Beijing Eye Study 2001/2006.

Cause	Frequency	Prevalence (%)	95% C I(%)
Cataract	68	24.9	19.8, 30.0
Glaucoma	23	8.4	5.1, 11.7
Diabetic Retinopathy	13	4.8	2.2, 7.3
Age-Related Macular Degeneration	10	3.7	1.4, 5.9
Myopic Retinopathy	9	3.3	1.2, 5.4
Macular Disease, Unclassified	5	1.8	0.2, 3.4
Epimacular Membrance	4	1.5	0.04, 2.9
Branch Retinal Vein Occlusion	2	0.7	0, 1.7
Non-Glaucomatous Optic Neuropathy	2	0.7	0, 1.7
Central Retinal Vein Occlusion	1	0.4	0, 1.1
Anterior Ischemic Optic Neuropathy	1	0.4	0, 1.1
Corneal Opacity	1	0.4	0, 1.1
Peripapillary Atrophy	1	0.4	0, 1.1
Unknown	133	48.7	42.8, 54.7

**Table 4:** Causes for incident visual field loss in the Beijing Eye Study 2001/2006 stratified by age.

Age (Years)	Causes	n	Frequency (%)	95%CI (%)
45–54	Cataract	1	9.1	0, 26.8
	Glaucoma	4	36.4	7.9, 64.8
	Other macular disease	3	27.3	1.0, 53.6
	Epimacular membrane	1	9.1	0, 26.8
	Anterior ischemic optic neuropathy	1	9.1	0, 26.8
	Non-glaucomatous optic neuropathy	1	9.1	0,26.8
	**Total**	11	100	
55–64	Cataract	13	50.0	30.8, 69.2
	Glaucoma	4	15.4	1.5, 29.3
	Diabetic retinopathy	3	11.5	0, 23.8
	High myopia	2	7.7	0, 17.9
	Age-related macular degeneration	1	3.8	0, 11.3
	Branch retinal vein occlusion	1	3.8	0, 11.3
	Optic neuropathy nonglaucoma	1	3.8	0, 11.3
	Corneal opacity	1	3.8	0, 11.3
	**Total**	26	100	
65–74	Cataract	33	49.3	0, 61.2
	Glaucoma	7	10.4	0, 17.8
	Diabetic retinopathy	10	14.9	0, 23.5
	High myopic retinopahy	6	9.0	2.1, 15.8
	Age-related macular degeneration	6	9.0	2.1, 15.8
	Other macular disease	1	1.5	0, 4.4
	Epimacular membrane	2	3.0	0, 7.1
	Branch retinal vein occlusion	1	1.5	0, 4.4
	Parapapillary atrophy	1	1.5	0, 4.4
	**Total**	67	100	
75+	Cataract	21	58.3	42.2, 74.4
	Glaucoma	8	22.2	8.6, 35.8
	High myopic retinopahy	1	2.8	0, 8.2
	Age-related macular degeneration	3	8.3	0, 17.4
	Other macular disease	1	2.8	0, 8.2
	Epimacular membrane	1	2.8	0, 8.2
	Central retinal vein occlusion	1	2.8	0, 8.2
	**Total**	36	100	

If a visual field defect was defined as moderate loss at any location as proposed by Kogure [36], incident VFL was found in 153 eyes (2.5%; 95%CI: 2.2%, 3.0%) or 138 participants (4.3%; 95%CI: 3.7%, 5.1%). It was associated with higher age (*P*<0.001), more myopic refractive error (*P*<0.001), higher systolic blood pressure (*P* = 0.004), higher intraocular pressure (*P* = 0.044), and higher fasting blood concentration of glucose (*P*<0.001). a visual field defect was defined as severe loss at any location, incident VFL was found in 34 eyes (0.6%; 95%CI: 0.4%, 0.8%) or 31 participants (1.0%; 95%CI: 0.7%, 1.4%). It was associated with age (*P*<0.001) only.

## Discussion

Few population-based studies have been performed so far focusing on the prevalence of visual field defects, and even fewer investigations addressed the new occurrence of VFL in a repeatedly examined population [22]–[25]. In our study on adult Chinese in Greater Beijing, the 5-year cumulative incidence of VFD was4.3±0.5% per eye or 7.3±0.9% per subject. For subjects with an age of 55 or more years at baseline, the incidence rate for VFL was 6.7±0.9% per eye or 11.5±1.6% per subject. A similar result was obtained in the population-based Rotterdam Study [23], which included 3761 persons whose age was 55 years or higher at baseline and who were free of visual field defects at baseline. After a mean follow-up time of 6.3 years, 175 (4.7%) persons developed a VFL [23]. The visual fields of both eyes were examined by suprathreshold perimetry, which was confirmed by Goldmann perimetry. The overall incidence rate of VFL in the Rotterdam Study was 7.4 per 1000 person-years, increasing to 21.1 per 1000 person-years in those aged 80 years and older. The incidence of VFL in our study was higher than that of the Rotterdam Study. The difference may be attributed to the different perimetric techniques and accordingly different definitions of a VFL. In the Rotterdam Study, a VFL was defined as the presence of a perimetric defect in at least one eye on Goldmann perimetry in a participant from the cohort at risk or the presence of a defect of at least 6 contiguous points on the last reliable supra-threshold perimetry performed at follow-up in those subjects for whom Goldmann perimetry was indicated but not performed. Frequency doubling threshold perimetry (program 20–1) was applied in our study and a VFL was defined as a reduced sensitivity at any location.

In all available studies on the incidence of VFL, the incidence and prevalence of VFL increased with age [13]–[25]. In our study, the five-year incidence of VFL was 1.2±0.2% per eye or 2.0±0.4% per subject for the age group of 40 to 49 years, and 10.6±1.3% per eye and 19.2±2.3% per subject for the age group of 70+years. This age-related increase in the incidence of VFL is paralleled by an age-related loss of retinal photoreceptors, retinal pigment epithelium cells and retinal ganglion cell axons of about 0.3% per year [37]–[39].

The association between the incidence of VFL and myopic refractive error can be explained by the morphologic changes characterizing myopic degenerative myopia [30]. The increase in the incidence of VFL with higher intraocular pressure is explained by the association between elevated intraocular pressure and glaucoma [40].

The major causes for incident VFL in our study were cataract, glaucoma and diabetic retinopathy, while age-related macular degeneration and retinal vein occlusion were less frequently responsible for an incident VFL. It reflects the list of disorders which were found to be the major causes for prevalence and incidence of visual impairment and blindness in population-based studies in China [2], [4]–[6], [16]. The latter studies focusing on visual acuity as well as our study addressing the incidence of VFL showed, that age-related macular degeneration is of lesser importance for presence and development of visual loss in adult Chinese in the Greater Beijing region and in Asians in general as compared with Westerners [16], [27], [41], [42]. In contrast to our study on Chinese, the Rotterdam Study revealed that glaucoma was overall the leading cause for incident VFD in the urban Dutch population of Rotterdam in all age categories [23]. In the Rotterdam Study, the overall incidence of glaucomatous VFD was 2.0 per 1000 person-years. In the same Rotterdam Study, stroke was the second most common cause of incident VFL in persons younger than 75 years, followed by age-related macular degeneration and retinal vascular occlusive disease.

The association between the incidence of VFL and fasting blood glucose level agrees with a previous study by Realini and colleagues who found that diabetic patients without retinopathy tended to perform poorly on FDT testing [43]. It shows that an abnormal frequency doubling technology testing in diabetic eyes may not represent glaucomatous visual field loss, and that the presence of diabetes, even in the absence of diabetic retinopathy, may reduce the specificity of frequency doubling technology in glaucoma screening.

Potential limitations of our study should be mentioned. First, the major concern, and a problem inherent to any epidemiologic study, is non-participation. The Beijing Eye Study 2006 had a reasonable response rate of overall 73%, 3251 of the 4439 subjects participated in the follow-up survey (or 61% of the group originally eligible in 2001); however, differences between participants and non-participants could have led to a selection artifact, and may have produced bias in the incidence rates of visual impairment and blindness. Second, 90.9% of the study population was younger than 70 years (40–69), 8.5% of the study population was 70–79 years old, and only 0.6% (19persons) was 80 years and older at baseline. The relatively low participation among the older, especially those older than 80 years, may also have led to an underestimation of the incidence of VFL. Third, another limitation of the present study may be the question how representative the rural regions and the urban regions of Greater Beijing are for the entire area of China. To get data on a purely rural region in China, far away from any major metropolitan region, the recent Handan Study from China may be more appropriate than the Beijing Eye study [4]. Fourth, in contrast to the Rotterdam Study [23], we did not have reliable information on the incidence of neurological diseases such as stroke and Alzheimer's disease as cause for incident VFL. It may partially explain why for almost half of the eyes with incident VFL, a cause for the VFL was not detected. Fifth, the visual field examinations were performed by frequency-doubling perimetry instead of by automated perimetry or Goldmann perimetry. Since the program (C-20-1) of the frequency-doubling perimetry used for this study was designed primarily for detection of glaucomatous visual field loss, and since the density of examination points in the visual field was relatively low, the study may have underestimated the prevalence and incidence of mid-peripheral or peripheral visual field defects., the reason for VFL was primarily determined by a single, however, experienced ophthalmologist, and in case of doubt, a panel decided about the reason for VFL. Although it would have been better, if two ophthalmologists had primarily decided about the reasons for VFL, the ophthalmologist was markedly experienced from previous studies to primarily determine the reason for VFL [16], [19], [27]–[30]. Seventh, one has to acknowledge that about half of the subjects with a VFL did not have any clearly detected cause related to the VFL. This may have been due to a learning effect (as has been described by Iester and colleagues [44]) since it was the first examination of this kind for the study participants. In addition, other definitions of a VFL could have been used such as by Iwase and coworkers [17] or Kogure [36]. Potential strengths of our study may be first that it is a population-based study with a relatively large sample size and a high participation rate (73%). Second, our study is one of the first investigations to look at incidence rates of VFL in a population from China, and it is one of the first studies on that topic worldwide.

In conclusion, the 5-year cumulative incidence of visual field loss in adult Chinese in Greater Beijing was 4.3±0.5% in eyes or 7.3±0.5% in subjects. It increased significantly with age, intraocular pressure, and fasting blood glucose concentrations. Major causes for incident VFL were cataract, glaucoma and diabetic retinopathy.
